# Orientation-Cue Invariant Population Responses to Contrast-Modulated and Phase-Reversed Contour Stimuli in Macaque V1 and V2

**DOI:** 10.1371/journal.pone.0106753

**Published:** 2014-09-04

**Authors:** Xu An, Hongliang Gong, Jiapeng Yin, Xiaochun Wang, Yanxia Pan, Xian Zhang, Yiliang Lu, Yupeng Yang, Zoltan Toth, Ingo Schiessl, Niall McLoughlin, Wei Wang

**Affiliations:** 1 Institute of Neuroscience, State Key Laboratory of Neuroscience and Key Laboratory of Primate Neurobiology, Shanghai Institutes for Biological Sciences, Chinese Academy of Sciences, Shanghai, P. R. China; 2 Key Laboratory of Brain Function and Diseases, School of Life Sciences, University of Science and Technology of China, Hefei, P. R. China; 3 Faculty of Life Science, University of Manchester, Manchester, United Kingdom; Monash University, Australia

## Abstract

Visual scenes can be readily decomposed into a variety of oriented components, the processing of which is vital for object segregation and recognition. In primate V1 and V2, most neurons have small spatio-temporal receptive fields responding selectively to oriented luminance contours (first order), while only a subgroup of neurons signal non-luminance defined contours (second order). So how is the orientation of second-order contours represented at the population level in macaque V1 and V2? Here we compared the population responses in macaque V1 and V2 to two types of second-order contour stimuli generated either by modulation of contrast or phase reversal with those to first-order contour stimuli. Using intrinsic signal optical imaging, we found that the orientation of second-order contour stimuli was represented invariantly in the orientation columns of both macaque V1 and V2. A physiologically constrained spatio-temporal energy model of V1 and V2 neuronal populations could reproduce all the recorded population responses. These findings suggest that, at the population level, the primate early visual system processes the orientation of second-order contours initially through a linear spatio-temporal filter mechanism. Our results of population responses to different second-order contour stimuli support the idea that the orientation maps in primate V1 and V2 can be described as a spatial-temporal energy map.

## Introduction

**Figure 1:**
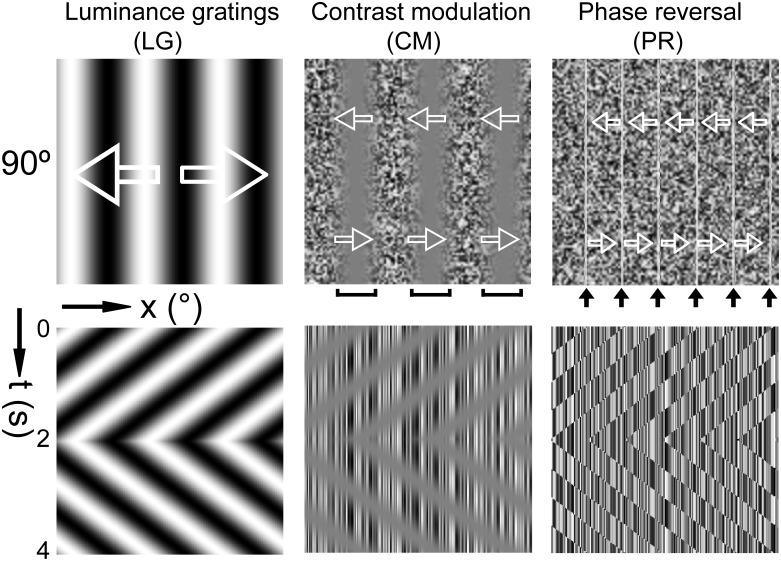
Synthetic first- and second-order contour stimuli. LG, sine-wave luminance gratings. CM, contrast modulated contours. PR, phase-reversal defined contours. Each column depicts one type of contour stimuli with an orientation of 90°. Arrows superimposed on each stimulus type in the top row represent the bidirectional motion of the global contours. The contours move leftward for 2 seconds and then rightward for another 2 seconds, as depicted below by the traces in the space-time plots. The square brackets and black arrows point to the second-order contours.

Visual perception arises from the transformation of neural signals along the visual hierarchy with neurons having different sizes of receptive fields (RFs) in each of its processing stages [1]–[2]. Humans and non-human primates can effortlessly see oriented contours or boundaries of objects, regardless of whether they are defined solely by a change in luminance (first order) or by contrast, texture, or other visual cues (second order). In contrast to a luminance defined first-order stimulus, all regions of a second-order stimulus contain the same average luminance (Fig. 1). Second-order stimuli were initially manifested by second-order motion as globally drift-balanced stimuli [3]–[4] and so it has been suggested that there exists separate visual channels specifically to process such stimuli [5]–[10]. General speaking, second-order stimuli reveal the dissociation between retinal inputs (Fourier components, first order) and visual percepts (non-Fourier features, second order).

It has been known for more than a half century that most neurons in early visual cortices have small spatio-temporal oriented RFs with precise retinotopic coordinates, exhibiting orientation selectivity to luminance-defined contours [11]–[19]. Therefore, our hypothesis is that the population responses in early visual cortices might be directly activated by the local luminance cues that define the global second-order contours. Specifically, we ask how is the orientation of second-order contours processed at the population level in macaque V1 and V2. This is an important question not only pertaining to the processing of orientation regardless of its defining cues (known as orientation-cue invariance) but also to the subsequent invariant representation of shapes and forms observed in the middle temporal (MT) area and V4 [20]–[26]. Population responses to contrast-modulated contour stimuli were previously found to be orientation-cue invariant in cat area 18 and a non-linear “filter-rectify-filter” model was subsequently proposed to account for this observation [9]. Recently, it was reported that neurons responding to contrast-defined contours in cat area 18 [8], [27] also encoded motion-defined second-order contours [28]. This is not the case in macaques as only a small number of cells in V1 and V2 were selective to the orientation of motion-defined contours [29]–[30]. A recent population study in macaque found that the preferences of population responses within V1 and V2 activated by illusory contour stimuli, that were defined by abutting lines, depended critically on the spatial frequency of the local carriers [25]. These results are compatible with a recent single-cell electrophysiological study, which demonstrated that most neurons in macaque V1 and V2 signal the orientation of first-order carriers within texture-defined herringbone patterns [31]. It appears that only a small number of neurons in the early visual cortices of non-human primate exhibit clear responses to second-order stimuli [23], [29], [32]–[37]. Thus, in this study we specifically investigated whether and how the orientation of second-order contours defined by contrast modulation and phase reversal is encoded by population responses in macaque V1 and V2.

We measured the cortical population responses to sine-wave luminance gratings (LG, first order), to contrast-modulated (CM) second-order contours, as well as to another set of second-order contours defined by phase reversal (PR) (Fig. 1), using intrinsic signal optical imaging of macaque V1 and V2. We found that contrast- and phase-defined contours both activated orientation domains with preferences in close register with those activated by first-order luminance-defined contours in V1 and V2, exhibiting orientation-cue invariance. In addition a physiologically constrained spatio-temporal energy model of V1 and V2 neuronal population responses was able to account for all the population responses recorded. Our experimental findings and energy model simulations suggest that the population responses in primate V1 and V2 reflect the spatio-temporal filter properties of orientation-selective neurons, and hence the orientation maps in the primate can also be described as a spatio-temporal energy map [38]–[41].

## Materials and Methods

### Ethics Statement

The experimental subjects were rhesus macaques (*Macaca mulatta*) and came from our institutional non-human primate breeding colony under licenses issued by National Forestry Ministry - Shanghai Bureau and Shanghai Animal Management Office. The animals were housed without cage in strict secure rooms with various environmental enrichment items, secure windows, and a balcony access to daylight. Specifically, the environmental enrichment items mainly included a large mirror on the wall, a swing, a rope for climbing, ladders, platforms, and an outdoor balcony for the animals to sunbathe and have outdoor activities (Fig. S1). The housing, husbandry, and breeding standards comply with National Laboratory Animal – Requirements of environment and housing facilities (GB: 14925-2010). All experimental procedures for primate research including animal euthanasia were approved by the Institute of Neuroscience Institutional Animal Care and Use Committee and by the local ethical review committee of the Shanghai Institutes for Biological Sciences.

### Animal surgical preparation and maintenance for optical imaging

A total of six adult male rhesus macaques each weighing 3.0∼4.5 kg were prepared and maintained for acute *in* *vivo* intrinsic signal optical imaging as described elsewhere [42]–[44]. In brief in each experiment, anesthetic induction was achieved by intra-muscular injection of ketamine hydrochloride (15 mg·kg^−1^, i.m.). After a tracheotomy, all surgical procedures were carried out under gaseous anesthesia (Isoflurane 0.5∼2% in 2∶1 N_2_O:O_2_). General anesthesia was maintained by bolus injections of Pentothal (sodium thiopental ∼2 mg/kg/h IV) administered by IV catheter. Depth of anesthesia was verified with respect to an electrocardiogram (ECG), pulse oximeter (SpO_2_), and end-tidal carbon dioxide (CO_2_), all of which were monitored continuously. A maintenance solution, of 5% glucose in saline, was administered by constant infusion through an intravenous catheter (3∼5 cm^3^/kg/h), whilst a thermister controlled electric blanket maintained the animal’s core temperature at around 38°C. The animal’s eyes were first washed with dH_2_O before the application of Atropine drops (1% solution) and the insertion of Plano hard gas-permeable contact lenses. Refractive errors were estimated using a slit ophthalmoscope and when necessary corrected using external lenses such that the animal focused on a screen placed 57 cm from the centre of its head. Craniotomy and durotomy were performed on both sides of the skull over V1 and V2 for dual optical imaging using two stainless steel chambers of 25 mm diameter secured to skull using dental cement. The lunate sulcus (LS) and superior temporal sulcus (STS) were used as cortical landmarks for surgeries. At the end of each experiment, euthanasia was achieved by the administration of a lethal IV injection of sodium pentobarbitone (50 mg). After death, the animal was immediately perfused transcardially with a saline rinse followed by ice-cold 1% paraformaldehyde in 0.1 M potassium phosphate buffer. The brain was then removed and the imaged visual cortices were flattened and sectioned for subsequent histological processing.

### Visual stimuli

A CRT monitor (Sony Trinitron Multiscan G520, 1280×960 pixels, 100 Hz) was placed 57 cm in front of the animal eyes extending 40×30 degrees as described elsewhere [25], [42]. The gamma of the monitor was carefully corrected by using the Color Calibration device (ColorCAL) from Cambridge VS system. Visual stimuli were computer-generated using custom software based on Psychtoolbox-3. For the contrast-modulated noise stimuli, the individual noise size of the noise carrier used in previous psychophysics and fMRI studies was between 1.81–13 arcmin [10], [45]–[54]. In this study, full screen noise textures were composed of noise elements randomly positioned with each element spanning approximately 5.4 arcmin. In some cases, we tested the noise size of 1.8 arcmin that corresponded to one pixel size on the CRT monitor, but we did not observe clear responses. We enlarged the noise size to 5.4 arcmin which activated reliable population responses across animals; therefore, we used this size in all cases. The luminance of the elements ranged from 0.2∼82 cd·m^−2^ following a uniform distribution. All stimuli employed had a mean luminance of approximately 45 cd·m^−2^. As illustrated in Figure 1, standard sine-wave luminance gratings (LG) were defined by the sequence of peaks and troughs where the contrast was defined as (Lmax−Lmin)/(Lmax+Lmin). Lmax and Lmin are the maximum and minimum luminance level of the stimuli, respectively. LG is regarded as a first-order stimulus (equation 1 and Movie S1). By comparison, the contrast-modulated grating was composed of noise texture whose pixels were modulated in contrast by a traveling sinusoid (equation 2 and Movie S2) and is called a second-order contrast-modulated (CM) stimulus [3], [8]–[9], [53], [55]–[57]. The appearance of CM stimulus is comparable to a traveling wave on the surface of water. In the texture phase reversed (PR) stimuli (equation 3 and Movie S3), second-order contours were generated from spatial locations where noise pixels had their polarity flipped simultaneously between frames. There is no change in either luminance or contrast in this stimulus type. Only the leading and trailing edges of the second-order contours are visible in the space-time plot (Fig. 1). The mean luminance in both first- and second-order stimuli was kept the same. The first- and second-order contours described above drifted back and forth perpendicular to their orientations for a total of 4 seconds, with 2 seconds for each moving direction, respectively (Fig. 1). The phase-reversal contours are dynamic second-order contours and disappear as soon as the stimuli stop moving. The top row in Figure 1 displays a single spatial frame (x, y) from each stimulus type with a space-time plot (x, t) below to aid the visualization of the movement.

For simplicity, we first define a two-dimensional moving sine wave as: 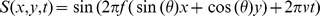, where *f* is the spatial frequency, 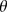 is the orientation, and *v* is the temporal frequency. Then the first- and second-order stimuli can be expressed as:
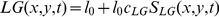
(1)

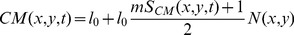
(2)

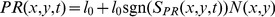
(3)Where *N(x,y)* is the uniform distribution [−1 1] for two dimensional noise texture, *l_0_* is the mean luminance (45 cd·m^−2^ in the experiment), contrast 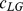 is set to 1, modulation depth *m* is set to 1, for LG, PR, and CM, the spatial frequency *f* and temporal frequency *v* were in the range of 1.0∼1.5 cycles per degree (cpd) and 4∼6 Hz.

### Optical imaging

Details of all the equipment and recording procedures of our custom built optical imaging system were as described elsewhere [25], [42], [58]. In brief, visual responses were recorded at sixteen frames per second for a period of 8 s, including 1 s prior to the stimulus onset under 630±10 nm red light illumination. The inter-stimulus interval was 13 s. Data were collected in an interleaved fashion for first- and second-order stimuli with different orientations. For first-order stimuli, data were typically averaged over 32 or 64 trials, while for second-order stimuli, the data were often averaged over 256 trials. The boundary of V1 and V2 was classically defined using either retinotopic space mapping or ocular dominance mapping [59].

### Optical image analysis

For each trial, frames taken between 3 and 7 s after the stimulus onset were averaged, and then subtracted and divided by a blank frame (the average response from the 1 s interval prior to the stimulus onset) to generate a map of reflectance change (ΔR/R map). Differential orientation maps were then created via pixel-by-pixel subtraction of reflectance maps generated by a pair of stimuli with orthogonal orientations (e.g. 0°–90°). Orientation preference maps were constructed using a vector summation algorithm [25], [42], [60]. We adopted a published method for removing pixels with large variability (e.g. those from blood vessels) and a mask was generated based on an objectively chosen threshold [9], [25], [42], [58]. Pixels covered by the mask were interpolated from surrounding un-occluded values just for display purposes but were never used in quantitative analysis. The interpolated images were then high-pass filtered (1.1∼1.2 mm in diameter) and smoothed (85∼323 µm in diameter) by circular averaging filters when necessary to suppress low and high frequency noise while avoiding signal distortion. Fractures of orientation preference map for LG were derived following Bonhoeffer and Grinvald (1993) [61], based upon a map of the magnitude of orientation gradient:
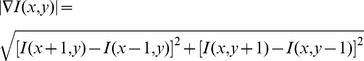
(4)where 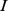 is the orientation angle value and 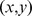 the pixel coordinates. Pinwheel centers of orientation preference map for LG were identified using a method similar to that of Crair et al. (1997) [62]: a pixel is considered to be a pinwheel center if the sum of orientation differences (wrapped into −90∼90°) between its four counterclockwise neighbors is ±180°. Previous and our current studies all found that pixels in pinwheel centers and fractures with rapid change of orientation preference were noise-sensitive and contributed disproportionately to the measurement variance in orientation preference maps [9], [63]–[65]. The pinwheel center pattern of an orientation preference map was dilated with a 5 pixel radius disk; its union with the fracture pattern (gradient of orientation change >15°/pixel) was used to form a binary mask to remove these noise-sensitive pixels from the comparison of different orientation preference maps [9]. Note that keeping these pixels in the calculation did not change the overall signature of the results. Using orientation preference maps obtained in an experiment with CM and LG stimuli as an example, the angular differences of orientation preference maps between first- and second-order contour stimuli that were less than 30° were 81% in both V1 and V2 if these pixels were kept in the calculation, while only slightly increased to 82% and 84%, respectively, in V1 and V2 after removing these pixels. For the quantification of the response amplitude, the max ΔR/R values in each responsive patch of a differential map were averaged, and this average intensity was taken as the response amplitude. A response profile analysis was performed in order to extract the orientation best represented by differential orientation maps [9], [25], [40], [42], [58], [66]. In brief, the signs of the ΔR/R values in the differential map were reversed, so that pixels responsive to the first condition of a trial had positive values while pixels responsive to the second condition exhibited negative values. The mean of all pixels was subtracted from each pixel thereafter. This ΔR/R map was then divided into 12 iso-orientation domains (0°−180°) based on the orientation preference map produced by LG stimuli, and the mean value of each domain was plotted as a function of its corresponding orientation. In addition, we adopted the spatial correlation coefficient (SCC) metric as an index of similarity between two differential maps that were evoked by first- and second-order stimuli (see Ramsden et al., 2001 for details). The value of SCC ranged between −1 and 1, with 1 indicating two identical maps while −1 indicating two identical but inverted maps. As in previous studies [67]–[68], coefficients within the range between −0.2 and 0.2 were deemed neither significantly overlapped nor segregated.

### Visual stimulus analysis

We analyzed the spectral power distributions of the first- and second-order stimuli in the frequency domain as follows:
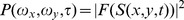
(5)where 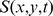 is the stimulus sequence, 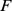 represents the Fourier transform (matlab function *fftn*), 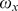 and 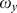 are the spatial frequency coordinates corresponding to the x and y directions respectively, 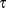 is the temporal frequency coordinate. To reveal the power distribution difference between the 90° and 0° oriented stimuli in the two-dimensional spatial-frequency space, the power distribution difference was integrated over all temporal frequencies:
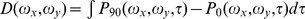
(6)the coordinate system of which can be converted to 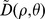, where 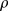 and 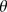 represent spatial frequency and orientation respectively. To show the differential power distribution in the individual spatial frequency, orientation, or temporal frequency dimension, the differential power was further integrated as follows:



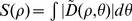
(7)

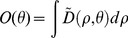
(8)

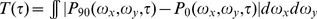
(9)


### Model simulations

We used the same model structure as that in our previous studies [42], [58], which was originally described by Mante and Carandini [41], [69]. Essentially, neuronal populations of both V1 and V2 were modeled as a bank of spatio-temporal filters and the average response of a set of neurons having the same tuning properties (orientation, spatial, and temporal preference) was given by integrating the energy of the stimulus falling into the receptive field (RF) of the population. To calculate the population responses of V1 and V2, here we further assumed that V1 and V2 are composed of neurons having a mixture of different tuning properties based on experimental observations [12]–[13], [70]. The spatial filtering property of neurons was modeled as a two-dimensional Gaussian function in frequency space:
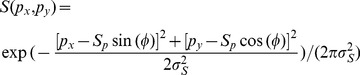
(10)where 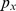 and 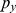 are the spatial frequency coordinates. 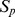 and 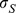 are the preferred spatial frequency and spatial bandwidth. 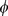 is the preferred orientation. Similarly, the temporal filtering property was modeled as a Gaussian function as follows:
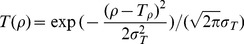
(11)in which 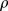 is the temporal frequency coordinate. 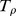 and 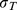 are the preferred temporal frequency and temporal bandwidth. Response of neuronal population with the same spatial frequency, temporal frequency, and orientation preferences was modeled as the integral over all spatio-temporal frequencies of the stimulus, scaled by the spatial and temporal filter functions:
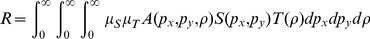
(12)where 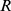 and 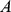 are the response and Fourier transformed stimulus, respectively. 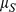 and 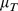 are two weights, corresponding to the proportion of neurons preferring the specific spatial and temporal frequencies respectively. In the simulations, preferred orientations (24 values) were uniformly distributed over 180°. Spatial frequencies (14 values) were logarithmically spaced between 0.13 and 11 cpd with a mean of 2.2 cpd for V1 [13] and 1.4 cpd for V2 [12], following Gaussian distributions which decided the weights 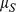. Temporal frequencies (16 values) were again logarithmically spaced between 0.25 to 45 Hz with a mean of 3.7 Hz for V1 and 3.5 Hz for V2 [13], following Gaussian distributions which decided the weights 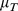. The spatial frequency and temporal frequency bandwidths were scaled to be 1/3 of the preferred spatial and temporal frequencies, as in Mante and Carandini [41]. For the simulation results, we averaged 256 trials as displayed on the monitor during experiments. We only analyzed a 15 by 15 degree sub-region of the full screen to reduce computation time, but the results were well approximated by this reduction. As in the optical imaging data, responses to two stimuli with orthogonal contour orientations were subtracted.

## Results

**Figure 2:**
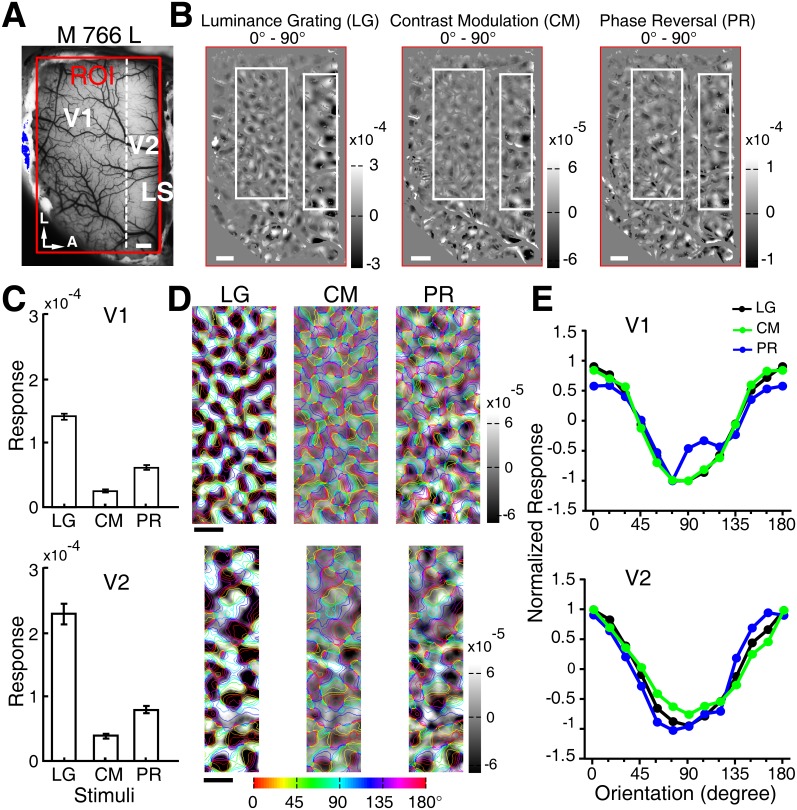
Orientation preference domains were activated by different first- and second-order contours in macaque V1 and V2. (A), Picture of the cortical surface taken from the left hemisphere of macaque 766 with region of interest (ROI) indicated by red box. This image was obtained under 550 nm green-light illumination. The broken white line indicates the border between V1 and V2. LS, lunate sulcus. L, lateral. A, anterior. (B), Differential orientation maps of 0° minus 90° in V1 and V2. Blood vessels were masked gray on all the maps. White boxes represent regions of V1 and V2 that were further analyzed and compared. (C), Response strength comparison for first- and second-order stimuli from ROIs of V1 and V2 in B. (D), Representative areas of V1 and V2 as boxed in B. Pixels covered by blood-vessel masks as shown in B were interpolated for clarity. Iso-orientation contours, derived from the orientation preference map generated using LG stimuli, were superimposed on each map. Colors of contours indicate orientation preferences as indicated by the color code below figure. (E), Normalized orientation response profiles for first- and second-order contour stimuli calculated from V1 and V2 areas in D. Error bars represent S.E.M. Scale bar: 1 mm.

We initially used second-order contours defined by carriers with small noise size corresponding to one pixel on the CRT monitor (about 1.8 arcmin). Perceptually the second-order contours can be clearly seen, however, we did not observe reliable and clear population responses to these stimuli in our optical imaging experiments of both V1 and V2. This may be because the SF components of these stimuli largely exceed the responsive range of the recorded areas. However all our second-order contour stimuli defined by noise size up to 5.4 arcmin elicited weak, but clear population responses in the recorded cortical regions of both V1 and V2 (Fig. 2).

### Orientation-invariant population responses activated by second-order contour stimuli

**Figure 3:**
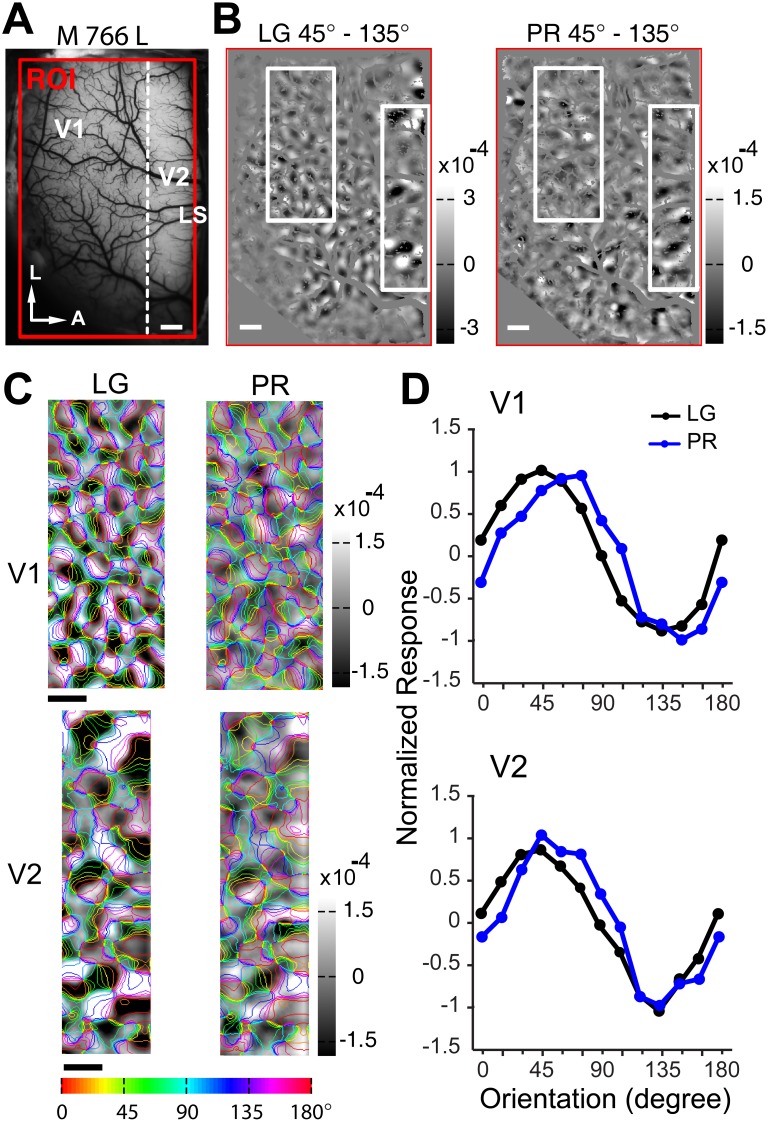
Orientation preference domains in V1 and V2 activated by phase-reversed contours. (A), Picture of the surface vasculature with the region of interest (ROI) in V1 and V2 as outlined by the red box. LS: lunate sulcus. A, anterior. L, lateral. (B), Differential orientation maps of V1 and V2 derived from LG and PR stimuli with orthogonal orientation pairs of 45° and 135°. Blood vessels were masked gray on all the maps. (C), Differential orientation maps from the representative areas of V1 and V2 (white boxes in B). Both pairs of the grayscale images were displayed based on the intensity range of the PR map and were superimposed with iso-orientation outlines derived from the orientation map generated by LG stimuli. (D), Normalized response profiles for LG and PR stimuli. The two pairs of curves were closely matched in the orientation preference for both V1 and V2. Scale bar: 1 mm.

**Figure 4:**
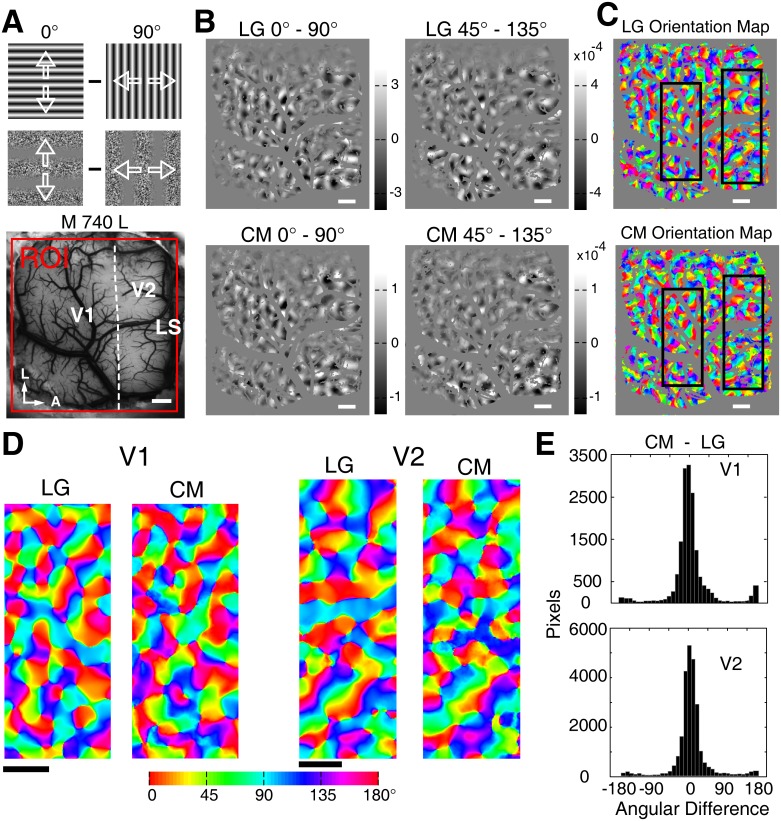
Orientation differential and preference maps in V1 and V2 for contrast modulated contour stimuli. (A), Picture of the surface vasculature of the left hemisphere of macaque 740 with the region of interest (ROI) in V1 and V2 outlined in red. Diagrams of the 0° and 90° oriented LG and CM stimuli were shown on top. (B), Differential orientation maps of V1 and V2 derived from LG and CM stimuli with orthogonal orientation pairs of 0°–90° and 45°–135°. Blood vessels were masked gray on all the maps. (C), Color coded orientation preference maps generated by LG and CM stimuli with blood vessels masked gray. (D), Orientation preference maps from representative areas of V1 and V2 as boxed in C. (E), Histograms produced by pixel-wise subtraction of the two pairs of orientation preference maps in D. The distributions of angular differences of preferred orientations peak around 0°. The percentages of pixels with angular differences less than 30° were 82*%* and 84*%* in V1 and V2, respectively. Note that orientation ranges from 0° to 180°, so the difference between two orientation values will be in the range of −180° to 180° by direct subtraction. Scale bar: 1 mm.

**Figure 5:**
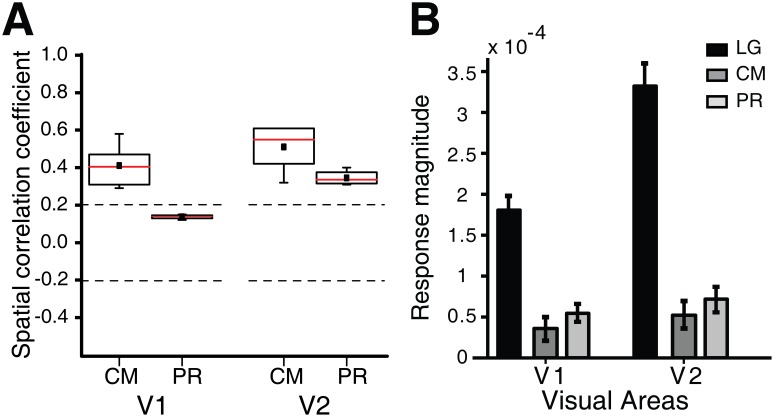
Spatial correlation coefficients (SCC) and response strengths across all animals studied. (A), Spatial correlation coefficients for both V1 and V2 between the first-order stimulus and the two second-order stimuli. SCC represents spatial similarity between the orientation differential maps activated by LG stimuli and those by CM and PR stimuli. The numbers of orientation differential maps computed were 6 and 4 for CM and PR stimuli respectively in both V1 and V2. The small solid black squares represent mean values while the red lines represent median values. (B), Comparison of population response strengths (ΔR/R) in V1 and V2. Error bars represent S.E.M. Data from 6 monkeys.

We generated differential maps of orientation preference within a region of interest (ROI) (Fig. 2A, B), by subtracting the intrinsic optical signals evoked by alternating full-field contour stimuli that had orthogonal orientations (0° and 90°). Dark regions prefer the first stimulus condition (0°) and bright regions prefer the second (90°). Within the same ROI of both V1 and V2, all contour stimuli activated orientation domains (Fig. 2B). However, responses to first-order stimuli were much stronger than those to second-order stimuli (Fig. 2C). Interestingly, all the stimuli evoked stronger responses in V2 than in V1. The response to luminance grating (LG) stimuli (1.40±0.04×10^−4^ in V1; 2.32±0.16×10^−4^ in V2) was nearly six times greater than that evoked by contrast-modulated (CM) stimuli (2.53±0.09×10^−5^ in V1; 4.00±0.27×10^−5^ in V2) and twice as large as that to phase-reversal (PR) stimuli (6.38±0.30×10^−5^ in V1; 7.97±0.52×10^−5^ in V2) (Fig. 2C). The standard error (SEM) was computed for the response from each black and white patch in the differential maps. For a direct comparison of the population responses activated by different stimuli, we presented the maps based on the gray-scale range of the population responses activated by CM (Fig. 2D). All comparisons were made between signals recorded from the same locations in V1 and V2. We performed response profile analysis [9], [25], [40], [42], [58] to determine the orientation preference of the functional domains activated by each of the oriented contour stimuli (Fig. 2E). As expected, we found that the response profiles produced by first-order contours closely matched the preferences of the underlying orientation columns in both V1 and V2. Interestingly, the functional domains activated by CM and PR stimuli were in close register with those evoked by the first-order contour stimuli of the same orientations. Data obtained with the same CM and PR stimuli yielded similar results in all animals studied (See Figs. 3 and 4 for further examples). By using a spatial correlation coefficient (SCC) index [25], [68], we also calculated the similarity between the differential orientation maps generated by LG and by CM and PR stimuli in V1 and V2 across all animals studied (Fig. 5A). The mean and median SCC values for CM and PR stimuli in both V1 and V2 were above or very close to 0.2, indicating significant overlap. The SCC value was just below 0.2 for the PR stimuli in V1. In addition, the SCC values for these two types of second-order stimuli in V2 were significantly higher than those in V1 (F(1, 16) = 13.22, *p*<0.01, two-way *ANOVA*), suggesting that V2 exhibits a higher degree of cue invariance than V1. Together, these results demonstrate that different types of second-order contour stimuli elicit clear population responses within orientation columns of both V1 and V2 in macaque. The orientation preferences of the population responses evoked by contrast-modulated and phase-reversed contour stimuli are closely matched to those activated by luminance gratings.

### Differences in response strength between first- and second-order contour stimuli

Figure 5B summarizes our findings on the average strength of responses induced by first and second-order contour stimuli across all six macaques studied. The relative change in the amount of light reflected (ΔR/R) from the ROIs in both V1 and V2 was measured and averaged for each stimulus type. We found that all the population responses to second-order contour stimuli (CM and PR) were significantly weaker than those to first-order LG stimuli in both V1 and V2 (F(2, 73) = 40.52, *p*<0.01, two-way *ANOVA*). This is most likely due to the fact that in contrast to sine-wave gratings the noise texture has a broadband power spectrum and as such contains energy at many different orientations. The contrast-modulated stimuli elicited the weakest responses, but were not significantly different from the phase-reversed stimuli (*p*>0.05, two-way *ANOVA*, post hoc Bonferroni test). In addition, the response to the first-order stimulus was significantly stronger in V2 than in V1 (*p*<0.05, two-way *ANOVA*, post hoc Bonferroni test). While the response strength to the second-order stimuli was also slightly higher in V2 than that in V1, this was not significant (*p*>0.05, two-way *ANOVA*, post hoc Bonferroni test). Altogether, these results imply that a similar mechanism may underlie the responses to these first- and second-order contour stimuli in both V1 and V2.

### Spatio-temporal energy model can account for the population responses recorded

Visual perception to global visual features or texture patterns is often modeled using a combination of linear and non-linear mechanisms [71]–[74]. The “filter-rectify-filter” (FRF) model [75], that was used to account for second-order motion perception, was also proposed to be responsible for neural responses to the orientation of contrast modulated second-order contours in cat area 18 [8]–[9]. However, the neural substrates corresponding to the different stages of the filter-rectify-filter model are still unclear [76].

**Figure 6:**
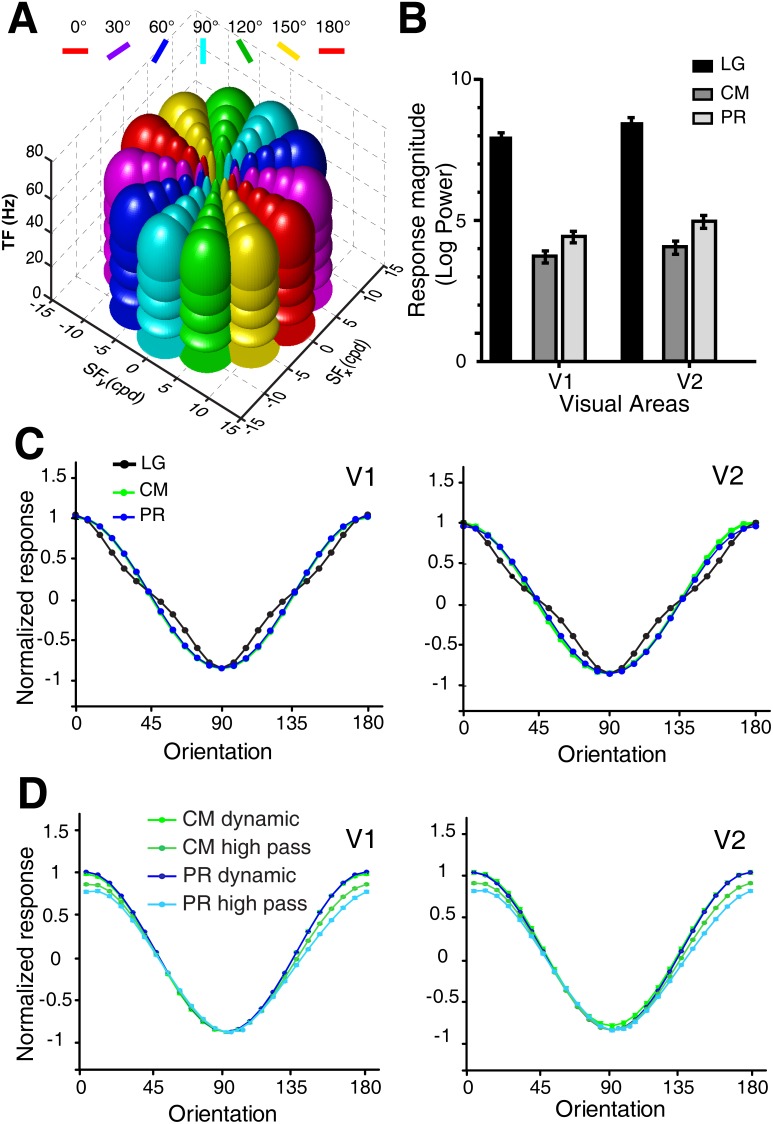
Energy model simulations of population responses to all stimulus types across V1 and V2. (A), Illustration of the receptive field of the modeled neuronal populations in the spatio-temporal frequency space. RFs with different tuning properties are assumed to be Gabor wavelets of different shapes, resulting in Gaussians in the frequency space, whose elliptic outlines are displayed. RFs illustrated in the same color correspond to a same orientation preference lying on radial sections in the frequency space (e.g. red: horizontal orientation; light blue vertical orientation). (B), Population response magnitudes produced by the energy model to different types of stimuli. (C), Normalized response profiles from V1 and V2 simulated by the energy model to the first- and second-order contour stimuli (0°–90°). As for the optical imaging experiment, the noise-texture carriers used for the simulation were static and were not filtered. (D), Normalized response profiles simulated by using dynamic and high-pass filtered (eliminate components with SFs below 9 cpd) noise textures as the carrier for CM and PR stimuli (0°–90°). The error bars indicate S.E.M over 256 trials and are smaller than the data marker.

In fact, the majority, if not all, of second-order stimuli contain both, global second-order features and local first-order luminance changes [9], [25], [31], [41], [55]–[56], [77]–[78]. For example, the PR and CM stimuli actually contain local luminance changes, as the luminance of pixels that define the boundary in the PR stimulus varies locally, so does the luminance of pixels that define the CM boundary (from black or white to mean grey). We thus examined whether a linear filter model could simulate our experimental results rather than the more complex “filter-rectify-filter” model suggested by an early study [9]. To do this we chose to implement a well-known spatio-temporal energy based model [79]. This spatio-temporal energy model has previously been employed to capture many response properties of early visual cortex at the single-cell level [80]–[83]. It has also been successfully applied to model the population responses of ferret visual area 17 elicited by different combinations of local visual features [38], [41]. More recently it has reproduced the population activities induced by motion axes of moving noise and dots in macaque V1 and V2 [42], [84] and in cat visual areas 17 and 18 [58]. The model of population responses is composed of spatial frequency, temporal frequency, and orientation filters with all parameters acquired from single-unit studies in macaque V1 and V2 [12]–[13], [70] (Fig. 6A).

We first simulated response strength to two orthogonal orientations (horizontal and vertical contours). Responses were derived from simulated population neurons of V1 and V2. The model produced weak responses to second-order contour stimuli (Fig. 6B) and the relative response strengths for all three types of stimuli were preserved (compare Figs. 5B and 6B). The ratios of response strength between LG and the rest of the stimuli were not perfectly matched between the model simulations and the optical imaging experiments. Two possible reasons may account for this discrepancy. First, the energy model did not take into consideration of a potential non-linear gain control mechanism within the circuits of orientation columns in V1 and V2 for the processing of second-order contours with low saliency. Secondly, a subgroup of specialized neurons within V1 and V2, which was previously reported to respond to second-order stimuli, contributed to the recorded population responses. Obviously this part of population response cannot be captured by a simple linear model. Nevertheless, the energy model predicted almost identical orientation response profiles for both first- and second-order (CM and PR) contour stimuli in V1 and V2 (Fig. 6C). It is known that dynamic and high-pass filtered noise textures can reduce the interference of first-order information in the processing of second-order features [6]. Besides static noise carrier, some psychophysical and fMRI studies also employed dynamic and filtered noise carriers [10], [47], [49], [52]–[54], [85]–[86]. Therefore, we also simulated the response profiles to CM and PR stimuli with dynamic and high-pass filtered noise carriers. The high-pass filtering of noise carriers eliminated components with SFs below 9 cpd that exceeded the optimal SF range of V1 and V2 neurons reported previously [13]. CM and PR stimuli formed by both dynamic and high-pass filtered noise carriers also evoked orientation cue-invariant population response in both V1 and V2 (Fig. 6D). This is consistent with the results obtained using static noise carriers.

**Figure 7:**
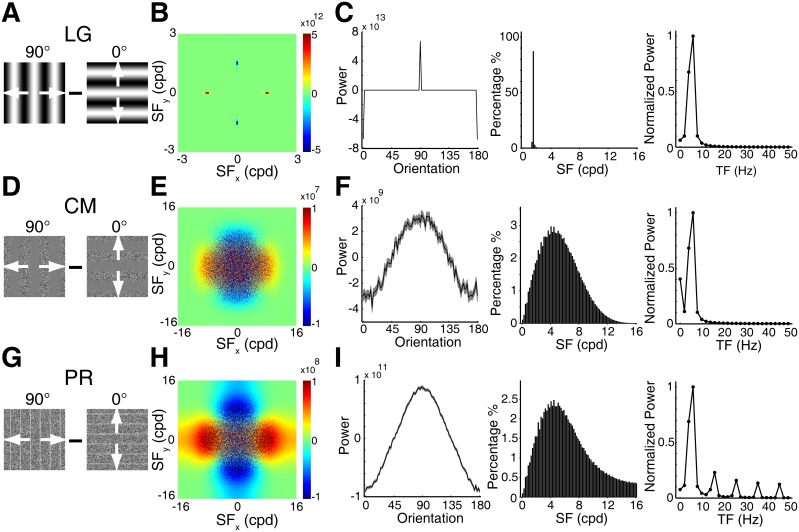
Distribution of differential power between stimuli with orthogonal orientations in the frequency space. (A, D, G), Diagrams of pairs of stimuli with orthogonal orientations of 90° and 0°. White arrows indicate the bidirectional motion of the stimuli. The stimuli moved 0.51 second in each direction to reduce the computational time. We then used a Fourier transform (matlab function ‘fftn’) to compute the power of each stimulus. (B, E, H), Power distribution difference (90°−0°) in the two dimensional spatial frequency space corresponding to each stimulus pair. Note that to reduce dimensions, the power was integrated over all temporal frequencies. 32 pairs of the CM and PR stimuli were used for the computation. (C, F, I), Differential power distributions in the orientation, spatial frequency, and temporal frequency dimensions for each stimulus pair, respectively. The gray shades in the left panels of F and I represent SEM over 32 pairs of stimuli.

A previous computational study has demonstrated that the analysis of the spectral power distributions in the frequency domain can reveal essential properties of second-order stimuli [78]. Our model results also can be intuitively understood through analyzing the first- and second-order stimuli in the frequency space (Fig. 7; see Materials and methods for details of the analysis). We firstly computed the power distribution difference between a pair of contour stimuli with orthogonal orientations (here using 90° and 0° orientations as examples) (Fig. 7A, D, and G). Then the distribution of the differential power in orientation, spatial frequency, and temporal frequency dimensions was analyzed. For the stimulus pair of luminance gratings, as expected, the differential power had a sharp peak and trough at 90° and 0°, respectively, in the orientation dimension (Fig. 7B, C). There were single peaks at ∼1.5 cpd and ∼5 Hz in the spatial and temporal frequency dimensions, respectively, corresponding to the spatial and temporal frequencies of the sine-wave gratings (Fig. 7C).

**Figure 8:**
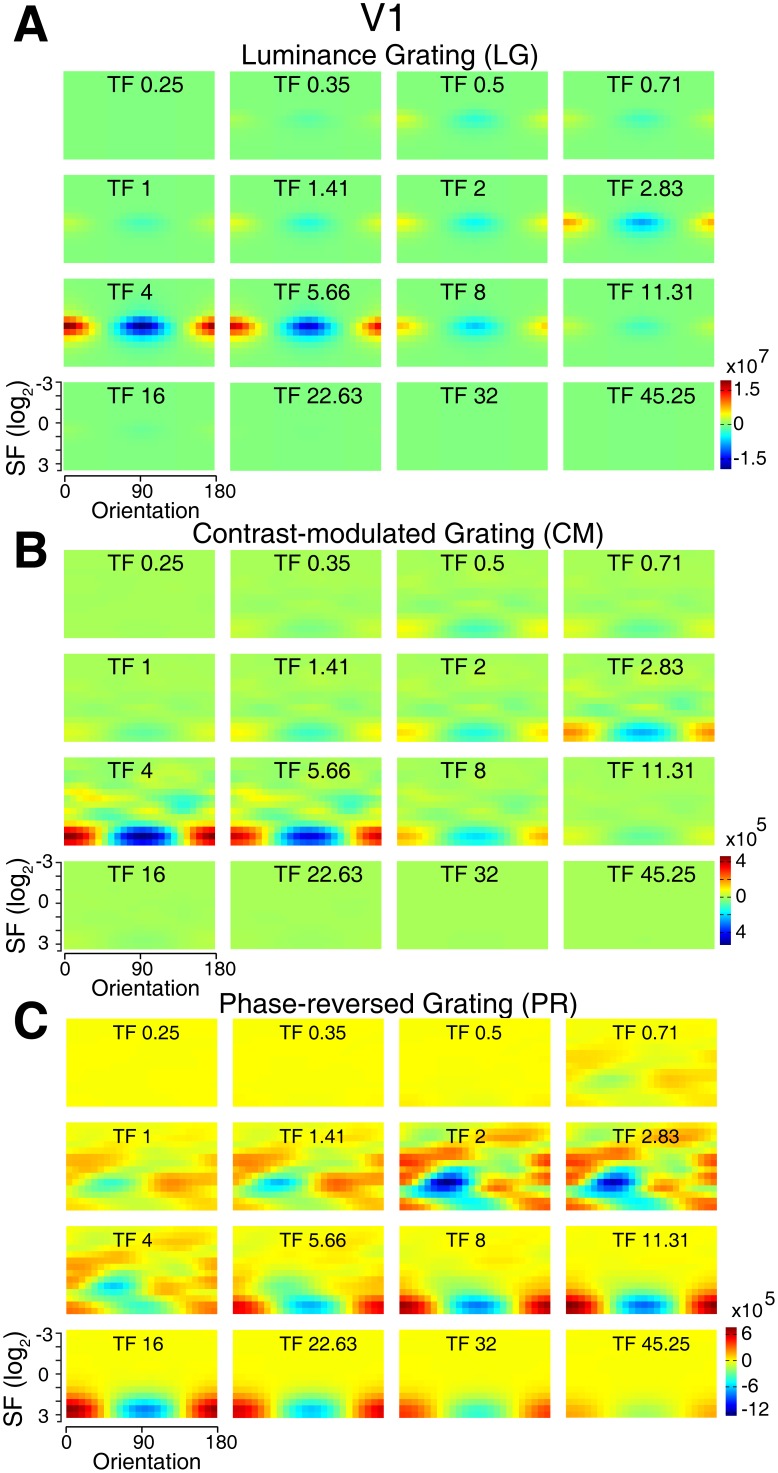
Simulated differential population responses with different preferences in V1. (A–C), Details of differential responses of neuronal populations preferring different orientations, spatial, and temporal frequencies to each stimulus pair (0°–90°). N = 10 trials.

**Figure 9:**
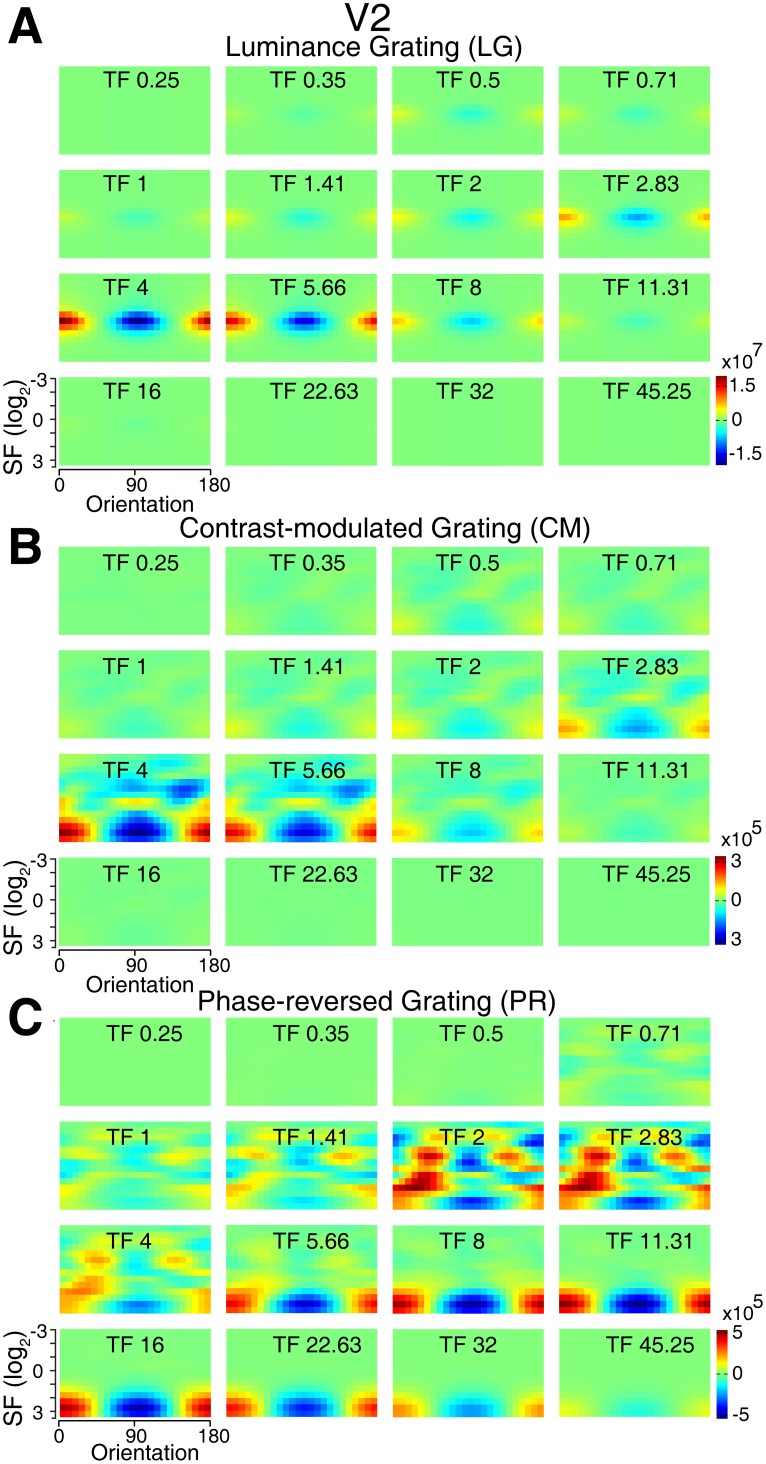
Simulated differential population responses with different preferences in V2. (A–C), Details of differential responses of neuronal populations preferring different orientations, spatial, and temporal frequencies to each stimulus pair (0°–90°). N = 10 trials.

We further examined how neuronal populations with different preferences to orientation, spatial, and temporal frequencies respond to the pair of 90° and 0° luminance gratings, and the results for V1 and V2 were presented in Figures 8A and 9A. The maximal differential population responses (0°–90°) occurred in the spatio-temporal frequency range corresponding to the stimulus parameters used for the pair of luminance gratings (Figs. 8A and 9A), demonstrating that model neurons are capable of reproducing the population responses activated by luminance gratings in both V1 and V2. In the case of the CM stimuli as the sinusoidal contrast modulation of a noise texture, this modulation causes a collinear distortion or inhomogeneous distribution in the local luminance of the broadband noise texture along the orientation of the contours (i.e. serrated edges). Thus the initial isotropic-noise structures within the noise texture tend to appear after modulation as oval shapes parallel to the contour orientation. This induced orientation signal can be clearly seen in the frequency space, as the differential power of the CM stimulus pair had a peak and trough at 90° and 0° respectively in the orientation dimension (Fig. 7E, F). Due to the broadband noise-texture carrier used, the differential power also existed in other orientations and distributed in a broad range of spatial frequencies (Fig. 7F). There was a sharp peak at ∼5 Hz in the temporal frequency dimension, corresponding to the temporal frequency of the stimuli (Fig. 7F). Thereby, the slight induced orientation signal is detected by the corresponding simulated neuronal populations, resulting in weak but consistent differential population responses selective to the orientation of the CM stimuli (Figs. 8B and 9B). Analogously, the response to the PR stimulus can be similarly explained by the fact that the PR stimulus is related to the CM stimulus, because it is in principle a noise texture modulated by a square wave (compare equations 2 and 3). Thus, also in this case, the modulation will introduce an anisotropic oriented component that was revealed by the analysis in the frequency domain (Fig. 7H, I). Noting that in comparison with the CM stimulus pair, the differential power of the PR stimulus pair spread to higher spatial and temporal frequencies (Fig. 7I) and this is caused by the square wave modulation. Similar to the CM stimuli, the power of the PR stimuli can be detected by the corresponding neuronal populations, leading to the orientation-cue invariant model responses (Figs. 8C and 9C). These results are comparable with recent studies on contrast-modulated second-order motion [46], [87]. In these studies, it was found that the texture contribution to second-order motion perception was mediated solely by the residual distortion products through the first-order mechanism and the non-existence of a separate second-order motion channel was consequently suggested. We further simulated the population responses to CM and PR stimuli with small noise size of 1.8 arcmin (corresponding to one pixel size on the CRT monitor) for the carrier. However, this condition generated the weakest response strength and no reliable and robust response profiles could be obtained, consistent with our experimental observations using optical imaging.

## Discussion

First- and second-order cues may occur spatially and temporally next to each other in natural scenes (often seen as texture) [8], [73]. The presence of local luminance fluctuations has been previously recognized within the synthetic contrast-modulated second-order stimulus [6], [55]–[56], [77]. This so-called luminance artifact cannot be completely avoided but only reduced. However, the second-order motion stimulus as a whole is drift-balanced without mean luminance changes [3] and the contrast modulated noise stimulus remains an important tool to study the processing of non-Fourier features [53], [55], [87]–[88]. Thus, noise texture has been widely used in human psychophysics and fMRI studies including those for making second-order motion stimuli. Similar to luminance cues in natural scenes or images, local contrast information has been suggested as an independent variable encoded by the early visual system [89]–[95]. Our findings suggest that it is these local first-order visual cues and related variations, which are collinearly distributed along the second-order contours, that drive the orientation cue-invariant population responses in macaque V1 and V2. This is also reflected in the observation that the population responses, activated by these second-order contour stimuli, are quite consistent between V1 and V2. This is because that macaque V2 not only receives its major feed-forward projections from V1 [96]–[97], but also responds to a similar range of spatial and temporal frequencies [12]–[13].

### The spatio-temporal oriented filter mechanism underlying population responses

In a natural setting, second-order contours are defined by a broad range of physical cues or components. In the laboratory, synthetic second-order contours can be defined by all sorts of first-order carriers regardless of their spatial components. To the second-order contour stimuli used in this study, a spatio-temporal energy model predicted all the orientation-selective responses (Figs. 6 and 8–9). These model results strongly suggest that it is the linear filter property of the small oriented spatio-temporal RFs of most V1 and V2 neurons that underlies the population responses. These population responses are consistent with an earlier observation in cat area 18, in which orientation-cue invariant population responses to contrast-modulated contour stimuli were also found [9]. By contrast, the population responses in cat area 18 were regarded as the responses to global second-order contours and a non-linear “filter-rectify-filter” model was subsequently proposed [9]. The main argument in this previous study was based on the hypothesis that the SFs of the carriers were above the cutoff SF of area 18; therefore, the population responses in the recorded region of area 18 should represent the non-linear neural responses to second-order contours. However, a recent study using electrophysiological single-unit recording of cat area 18 and monkey V1 and V2 found very few neurons responding to pure second-order visual features [31]. In fact, the responses of most neurons greatly decreased when the spatial frequency of the carriers was set beyond the sensitive range of the neurons. In the current study of macaque V1 and V2, we also did not observe reliable population responses when the individual noise element of the noise carrier was set to 1.8 arcmin corresponding to high carrier spatial frequency.

It is also important to note that the stimuli were not presented exclusively within a restricted visual field corresponding to the recorded cortical region, but instead presented across the full screen that activated a large section of the retina including central vision. Thus, another interesting question arises as whether neurons having RFs at different eccentricities within different regions of V1 and V2 employ different mechanisms for the processing of the same second-order contour stimulus. Specifically, do neurons in the region corresponding to the central visual field of high spatial resolution utilize linear mechanisms, while neurons representing peripheral space, with low spatial resolution utilize non-linear mechanisms? Together, it is conceivable that for the processing of contrast-modulated contours defined by noise carriers with different SFs, cue-invariant orientation responses may be produced by different neuronal mechanisms in early visual cortices. However, this requires further investigations, particularly when considering the fact that orientation is represented in both V1 and V2 with spatial frequency invariance.

Using the spatio-temporal energy model, but with the receptive-field parameters of neurons for V1 and V2, we were able to reproduce the previous observation of orientation-cue invariant population responses to different texture-defined or contrast-modulated contour stimuli. This result suggests that the “filter-rectify-filter” model is not essential to account for the population responses recorded in macaque V1 and V2. Interestingly, it was previously found that the orientation preferences of population responses evoked by illusory contours were always orthogonal to those activated by luminance gratings not only in cat areas 17 and 18 but also in macaque V1 and V2 [25], [68], [98]. Furthermore, the linear energy model could reproduce population responses with an orthogonal orientation preference evoked by illusory-contour stimuli in both species, but not the partially shifted orientation preference observed in monkey V1 and V2 [25]. This suggests that the processing of second-order contours created by modulating noise carriers are different from that of other type illusory or filling-in figures, which are more complicated and clearly need to employ not only separate non-linear integrative mechanisms but also cortical feedback [25], [34], [99]–[101].

The processing of local first-order visual cues has been previously observed and predicted for the neuronal firings to other stimuli of texture-defined patterns or forms in macaque V2 [31], [102]–[103]. Psychophysical studies have also suggested that spatial and temporal pooling of orientation-selective filters is a fundamental aspect of the low-level visual processing that underlies orientation discrimination [104]–[106]. Similar first-stage filters for orientation and spatial frequency have been proposed to sub-serve a common mechanism for the detection of contrast- and texture-defined boundaries [31], [46], [73], [87]–[88]. Furthermore, results from numerous psychophysical studies suggest that arrays of V1 neurons may integrate their responses to local components into global contours when the local components are collinearly aligned [107]–[112]. This idea is reinforced by anatomical studies investigating lateral and horizontal connections and population responses of V1 [113]–[118]. However, at the levels of columns and circuits particularly for generating the visual responses of a subgroup of specialized neurons previously reported to signal second-order stimuli [27], [29], [32]–[37], the actual neuronal mechanisms could be much more complicated. So is the case for the processing of the direction signal of second-order motion, which was proposed to invoke a separate non-linear visual mechanism [3], [6]–[7], [53], [56], [78], [119]–[120].

### Non-linear integrative processing for second-order contours

The spatio-temporal energy model can only capture the linear aspect of neural population responses in V1 and V2 to our second-order contour stimuli. However, visual non-linearities start with phototransduction within the retina. Indeed, nonlinear spatial integration and fine-scale heterogeneities in spatial sampling for ganglion cells of mouse retina have been revealed recently [121]. Similarly, responses to some second-order cues have been demonstrated as early as in the guinea pig retina and thalamic Y-type cells in cats [122]–[124]. Thus, it may not be surprising that a small percentage of high-order neurons within V1 and V2 of non-human primates respond to non-luminance defined boundaries [23], [29], [32], [37], [125]. It is impossible to extract the contribution of this small group of neurons from our recordings as our responses are derived from pooled neuronal activities. It is also worth noting that the weak intrinsic signals generated by second-order stimuli in this study are not consistent with the subjective experience when viewing the stimuli. We readily perceive all these second-order contours without any extra effort and largely independently of the carriers. This suggests that neuronal mechanisms, particularly those underlying response gain control or normalization and retinotopic spatial pooling, involve non-linearities in visual cortices [25]–[26], [73]–[74], [83], [126]. The contribution of surround suppression to encode second-order features at multiple stages may also need to be considered [86], [127]. Previous studies have found that the feedback from higher visual cortices can enhance neuronal responses in V1 to higher-order visual stimuli of low saliency [128]–[129]. This interaction between low- and high-level visual areas also needs to be considered [130]–[135]. Thus, it appears that the perception of second-order stimuli engages the hierarchical processing from early visual cortices to high brain stages with different focuses in each processing stage. Regardless of the mechanisms involved, the spatio-temporal filtering mechanism revealed here by energy model at the population level in macaque V1 and V2, constitutes the foundation for processing the orientation of second-order contours defined specifically by modulating noise-texture carriers.

### Conclusions

It is well-known that the complexity of encoded visual features increases greatly from V1 to V4 and IT, consistent with the great increase of RF size along the visual hierarchy [1]–[2], [136]–[137]. Therefore, it is likely that at the population level the higher processing stages may capture different features from second-order contour stimuli [26], [138]–[140]. In contrast, the small spatio-temporal RFs of the V1 and V2 neurons are more suitable to the detection of the local luminance variations in these stimuli such as the serrated edges produced by the white and black pixels. It is also known that foveal and parafoveal RFs in primate V1 and V2 are quite small and have super-high spatial resolution. The grating acuity is >26 cpd in monkey [141] and that is higher than the SFs of stimuli used in the current and most if not all previous studies on second-order visual processing. Thus, population activities in early visual cortices of V1 and V2 mainly reflect the processing of local luminance changes while those in higher visual areas represent global second-order features. Our experimental results demonstrate that the orientation of contrast-modulated and phase-reversed contours is invariantly represented in the population responses of macaque V1 and V2. Simulations based on a physiologically constrained energy model further suggest that the spatio-temporal filter mechanism of the majority of V1 and V2 neurons underlies the recorded population responses. Our findings also suggest that the orientation maps in macaque V1 and V2 can be described as a spatio-temporal energy map [40].

## Supporting Information

Figure S1 **The inside and outside view of our non-human primate housing facilities.**
(TIF)

Movie S1 **An illustrative movie shows sine-wave luminance gratings of 0° and 90° orientations.**
(AVI)

Movie S2 **An illustrative movie shows contrast-modulated noise stimuli of 0° and 90° orientations.**
(AVI)

Movie S3 **An illustrative movie shows second-order contour stimuli of 0° and 90° orientations defined by the phase reversal of noise texture.**
(AVI)
