# Supplementary figures and images for: Orientation-Cue Invariant Population Responses to Contrast-Modulated and Phase-Reversed Contour Stimuli in Macaque V1 and V2
Source: PLoS One. 2014 Sep 4;9(9):e106753. doi: 10.1371/journal.pone.0106753 (PMC4154761; doi:10.1371/journal.pone.0106753)

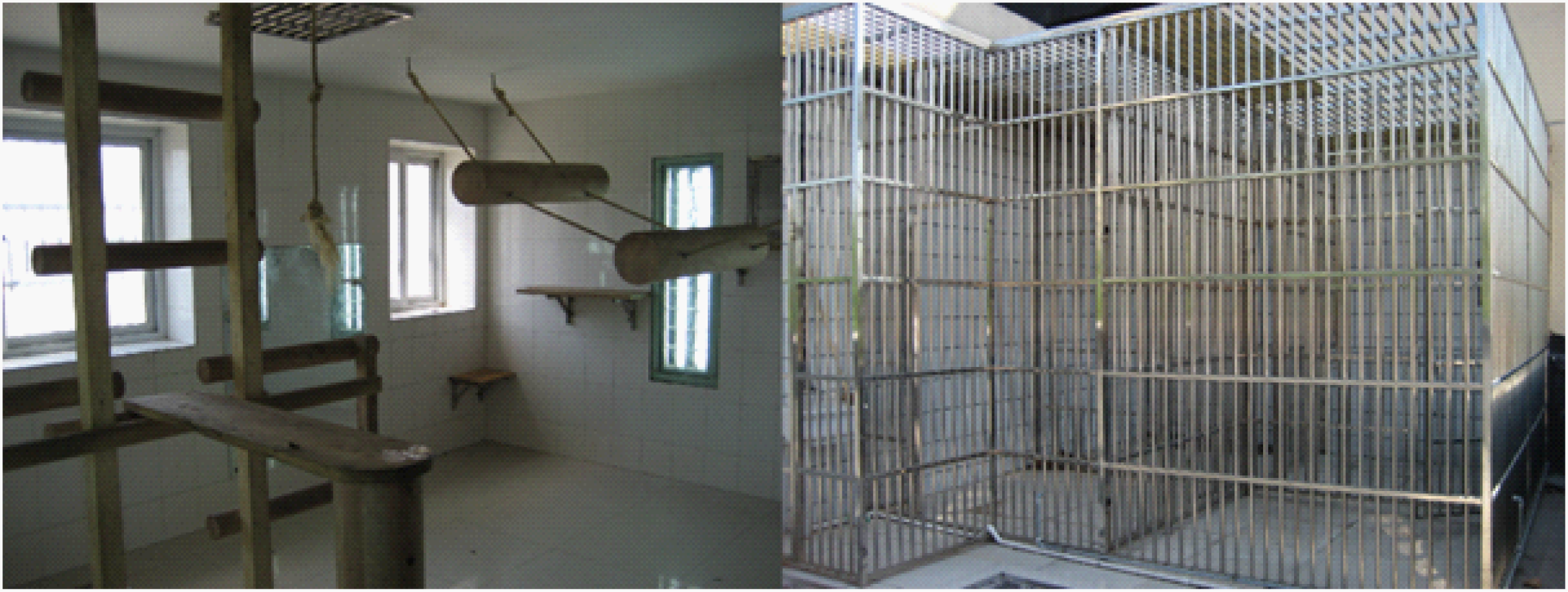

Supplement: Figure S1 — The inside and outside view of our non-human primate housing facilities. (TIF) [file pone.0106753.s001.tif]
